# Anticancer LNA Oligonucleotides Detection through a Simple Paper-Based Platform

**DOI:** 10.1021/acsmeasuresciau.5c00164

**Published:** 2026-02-10

**Authors:** Ada Raucci, Giovanna Liciberto, Michele Guida, Thomas Lee Moore, Canio Martinelli, Michelino De Laurentiis, Antonio Giordano, Stefano Cinti

**Affiliations:** † University of Naples Federico II, 9307 Department of Pharmacy, Via D. Montesano 49, Naples 80131, Italy; ‡ Sbarro Institute for Cancer Research and Molecular Medicine, Center for Biotechnology, College of Science and Technology, 6558 Temple University, Philadelphia, Pennsylvania 19122, United States; § Department of Human Pathology of Adult and Childhood “Gaetano Barresi”, Unit of Obstetrics and Gynecology, University of Messina, Via Consolare Valeria 1, Messina 98124, Italy; ∥ Department of Breast and Thoracic Oncology, Istituto Nazionale Tumori IRCCS “Fondazione G. Pascale”, Napoli 80131, Italy; ⊥ Department of Medical Biotechnologies, University of Siena, Siena 53100, Italy; # Department of Chemistry, Faculty of Science, Chulalongkorn University, Bangkok 10330, Thailand

**Keywords:** LNAs, electrochemical biosensors, miRNAs, antisense oligonucleotides, personalized medicine

## Abstract

Locked nucleic acids (LNAs) are chemically modified oligonucleotides widely used in gene silencing and precision oncology, yet their clinical translation is limited by the absence of simple, rapid assays to quantify them directly in biological fluids for dose optimization and timely therapeutic feedback. Here, we report a paper-based electrochemical biosensor for the direct detection of antisense LNA oligonucleotides using LNA-anti-miR-155 as a clinically relevant model. The platform employs a methylene blue-labeled RNA probe that mimics the native miRNA target and yield a signal-off voltammetric response upon hybridization. The disposable, gold nanoparticle-modified paper electrodes support picomolar detection limits, high reproducibility, and reliable performance in undiluted human plasma without sample pretreatment. By combining portability, low cost, and rapid analysis in a single-use format, this biosensor advances point-of-care monitoring of LNA-based therapeutics and provides a versatile blueprint for future personalized treatment platforms targeting antisense oligonucleotides.

## Graphical abstract

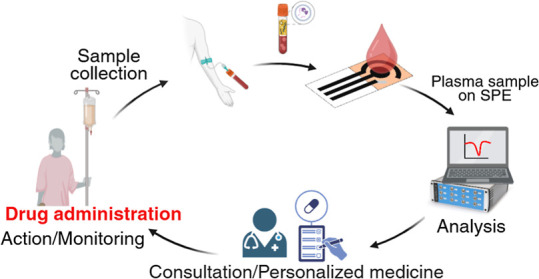

## Introduction

Synthetic oligonucleotides have emerged as powerful tools for modulating gene expression, revolutionizing precision medicine in oncology and chronic inflammatory diseases. Among them, locked nucleic acids (LNAs) are particularly attractive due to their methylene-bridged ribose, which enhances base-pairing affinity, thermal stability, and resistance to nuclease degradation.^1^ Structurally, LNAs are ribose-modified analogues in which the sugar ring is “locked” by an O2′–C4′ methylene bridge into an RNA-like C3′-endo conformation, enforcing an A-form duplex and markedly increasing affinity for complementary RNA; duplexes incorporating LNA monomers typically display melting-temperature gains of about +2 to +10 °C per residue relative to the corresponding DNA/RNA hybrids, together with enhanced nuclease resistance and preserved Watson–Crick specificity.^2,3^ LNAs are widely employed as antisense inhibitors of microRNAs (miRNAs),^4^ small noncoding RNAs that regulate critical oncogenic and immunological pathways^5,6^ and by forming stable heteroduplexes or inducing RNase-H-mediated cleavage, LNA-antimiRs effectively silence target miRNAs,^7^ as demonstrated by Miravirsen, which targets miR-122 in liver disease, and Cobomarsen, which inhibits the oncogenic miR-155.^8–10^ These examples highlight the growing therapeutic relevance of LNA-based strategies and the need for analytical tools to monitor their pharmacokinetics in real time.

Conventional methods for LNA detection, such as high-performance liquid chromatography, tandem mass spectrometry, or qPCR,^11–13^ offer high accuracy but require sophisticated infrastructure, long processing times, and are incompatible with point-of-care applications.^14^ In contrast, electrochemical biosensors combine high sensitivity, low cost, and portability with the ability to operate in unprocessed biological matrices.^15,16^ Their performance relies on selective hybridization at the biorecognition interface, where a synthetic RNA probe mimicking the native miRNA selectively captures the complementary LNA antisense strand.

Despite these advantages, no electrochemical biosensor has been specifically reported for the direct detection of intact therapeutic LNAs in plasma. Electrochemical nucleic acid biosensors reported to date have predominantly targeted endogenous miRNA or DNA biomarkers and are typically implemented on planar or microfabricated electrodes operated in buffer or partially processed biological samples. In parallel, paper-based electrochemical devices have emerged as low-cost, disposable platforms for on-site bioanalysis, but they are still mainly applied to native biomarkers or model sequences and usually require diluted or pretreated matrices rather than undiluted plasma.^17–19^ To the best of our knowledge, no electrochemical biosensor has been described that directly measures intact antisense LNA oligonucleotides in undiluted human plasma on a single-use paper strip without enzymatic amplification or sample pretreatment. Building on these advances, we developed a paper-based electrochemical biosensor for rapid detection of antisense LNA oligonucleotides. As a proof of concept, we selected LNA-anti-miR-155, a clinically relevant inhibitor of miR-155, and immobilized a methylene blue (MB)-labeled RNA probe mimicking miR-155 onto disposable gold nanoparticle-modified electrodes. The device generates a clear signal-off response upon LNA hybridization and operates directly in undiluted human plasma without enzymatic amplification or sample pretreatment. While this sensing architecture builds upon well-established redox-tag hybridization designs (e.g., E-DNA platforms),^20^ its application to detect therapeutic LNA oligonucleotides directly in untreated human plasma has not been previously demonstrated on a low-cost, portable platform. The assay operates in a single step at the point of use without requiring added enzymes, secondary labels, or external reporters, making it fully reagentless and suitable for point-of-care monitoring of LNA therapeutics.

## Experimental Section

### Materials and Methods

All reagents used were of the highest quality available. PBS tablets (140 mM NaCl, 10 mM phosphate buffer, 3 mM KCl), Chloroauric acid (HAuCl_4_), sodium borohydride, sodium citrate, sodium chloride (NaCl), *tris*(2-carboxyethyl) phosphine (TCEP; C_9_H_15_O_6_P) and 6-Mercapto-1-hexanol (MCH, C_6_H_14_OS), and human plasma were purchased from Sigma-Aldrich (St. Louis, MO, USA). The oligonucleotide probes, including miR-155 DNA–DNA Oligo (5′-Thiol- C6-TTA ATG CTA ATC GTG ATA GGG GT-Atto MB2-3′), miR-155 RNA–RNA Oligo (5′-Thiol- C6- uua aug cua auc gug aua ggg gu-Atto MB2-3′), anti-miR-155 RNA–RNA Oligo (5′-acc ccu auc acg auu agc auu aa -3′) and anti-miR-155 LNA 50% B-LNA Oligo (5′- (+A)­(+C)­(+C)­(+C)­(+C)­(+T)­(+A)­(+T)­(+C)­(+A)­(+C)­G ATT AGC ATT AA -3′) were obtained from Metabion GmbH (Steinkirchen, Germany). Sequences tested as potential interferents, including miRNA-21 (5′-uag cuu auc aga cug aug uug-3′), miRNA-218 (5′-uug ugc auc uaa cca ugu-3′), miRNA-29c-5p (5′-uga ccg auu ucu ccu ggu guu-3′), miRNA-101-5p (5′-cag uua uca cag ugc uga ugc u-3′) and the anti-miR-644a ASO LNA (5′-AGT (+G)­(+T)­(+G)­(+G)­(+C)­(+T)­(+T)­(+T)­(+C)­(+T)­(+T)­(+A)­(+G)­AG C-3′) were also sourced from Metabion GmbH (Steinkirchen, Germany). Adobe Illustrator was used to draw the wax model of the creation of the hydrophilic test area on the filter-paper electrodes. A solid ink printer, the ColorQube 8580 from Xerox (USA), was used to print the hydrophobic wax layer. All the electrochemical measurements were carried out using a portable potentiostat PalmSens 4 (PalmSens, Netherlands) equipped with a multi-12 reader and interfaced to a laptop using PSTrace5.9. All potentials reported are referred to the Ag/AgCl pseudo reference of the screen-printed electrochemical strips. GloMax Explorer Multimode Microplate Reader (Promega, USA) was used to quantify LNA concentration through the Quant-iT RiboGreen fluorescence assay.

### Paper-Based Screen-Printed Electrode Preparation and AuNPs Synthesis

All this information is reported in the Supporting Information.

### Preparation of the Specific Device for the Determination of LNA-Anti-miR-155

To fabricate the biosensor, the first step was to modify the surface of the working electrode by applying 4 μL of an AuNPs suspension by drop-casting. The presence of gold on the electrode is essential for the subsequent immobilization of the recognition element. The capture probe employed in this setup was a synthetic RNA oligonucleotide (5′-Thiol-C6-UUA AUG CUA AUC GUG AUA GGG GU-AttoMB2-3′) designed to mimic the native target of miRNA-155. This probe had a thiol group at the 5′ end, which allowed a strong covalent bond with the AuNP-modified surface through the Au–S bond. Prior to immobilization, the thiol moiety was activated by reduction with 10 mM *tris*(2-carboxyethyl)­phosphine (TCEP) for 1 h at room temperature to ensure complete deprotection. After reduction, the probe was diluted to nanomolar concentrations (500, 250, 100, and 50 nM for the preliminary optimization experiments, and a final concentration of 100 nM was selected for electrode functionalization based on the results shown in Figure S2B), and 20 μL of the solution was applied to the AuNPs-coated electrode area. The hybridization layer was allowed to form by passive adsorption during a 1 h incubation at room temperature. Subsequently, the electrodes were gently rinsed with ultrapure water to remove unbound material. To minimize nonspecific adsorption and improve the uniformity of the monolayer, the electrode surface was further treated with 20 μL of 2 mM 6-mercapto-1-hexanol (MCH) and incubated for 1.5 h at room temperature. This coassembly of thiolated RNA and MCH forms a compact, hydrophilic layer on the AuNP-modified gold surface that helps limit fouling by plasma components while preserving probe accessibility. A final rinse with double-distilled water was performed to remove any excess reagent and ensure a clean, passivated surface. All functionalization steps (probe immobilization and MCH backfilling) were performed in a closed Petri dish containing a water-soaked tissue to maintain a humid environment, with the lid kept closed during incubation to prevent solvent evaporation and preserve reaction uniformity.

### Experimental Procedure

The detection platform was designed to link the decrease in MB-based current to the increase in concentrations of the target LNA-anti-miR-155, following a typical signal-off mechanism. This behavior is attributed to a conformational change that occurs during hybridization between the recognition probe and the LNA sequence, which leads to a more rigid structure and reduces electron exchange between MB and the electrode surface. Specifically, when LNA-anti-miR-155 is absent, the probe remains in a flexible conformation, allowing the MB tag to interact closely with the electrode, thereby promoting electron transfer and generating a high electrochemical signal. Conversely, in the presence of the target, the probe binds to the complementary LNA sequence, giving rise to a more structured duplex that distances MB from the electrode, limiting the Faradaic transfer of electrons. As a result, the current signal decreases in proportion to the concentration of LNA in the sample. The combination of a flexible MB-labeled RNA probe on an AuNP-enhanced, MCH-passivated surface, together with SWV readout, provides a distance-dependent signal-off response with sufficient gain and low noise to achieve subnanomolar sensitivity in plasma. The electrochemical responses were recorded after a 30 min incubation using square wave voltammetry (SWV) as an electrochemical technique (equilibrium time = 5 s, *E*_start = 0.0 V, *E*_end = −0.6 V, *E*_step = 0.001 V, amplitude = 0.01 V, frequency = 25.0 Hz). Measurements were performed on 12 SPEs in parallel, using a PalmSens 4 portable potentiostat connected to a 12-channel multiplexer. The percentage change in signal (signal change %) was calculated according to the equation: signal change % = (*I*_0_ – *I*_target_)/*I*_0_ × 100, where *I*_0_ is the current measured in the absence of the target and *I*_target_ is the current measured after target hybridization, as shown in Figure 1.

**Figure 1:**
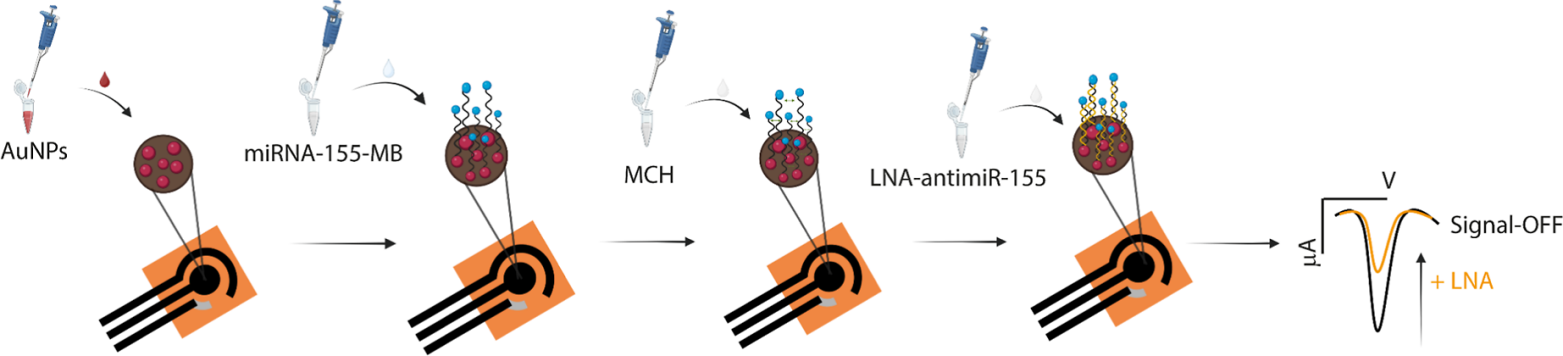
Schematic representation for the development of the electrochemical biosensor for the detection of LNA-anti-miR-155 and the expected current signal in the absence and presence of target.

This procedure was applied consistently in both standard buffer samples and human plasma samples, confirming the reliability and sensitivity of the biosensor even in complex biological conditions.

## Results and Discussion

### Evaluation of the Experimental Setup

The development of the electrochemical biosensor for the detection of LNA-anti-miR-155 required a systematic evaluation of each experimental parameter to ensure sensitivity, reproducibility, and stability in both buffer and complex biological matrices. In this study, the surface chemistry and electrochemical readout build on established hybridization-based architectures, but the novelty lies in adapting this simple design to the direct detection of intact antisense LNA therapeutics in undiluted human plasma on a single-use paper strip, rather than to model DNA or miRNA targets in buffer on rigid electrodes. The detection interface was built on SPEs modified with AuNPs, which provided a large surface area for probe immobilization and facilitated efficient electron transfer. The optimization of the experimental parameters was performed in a standard phosphate buffered solution (pH 7.4) in order to obtain satisfactory analytical performance toward the target.

The first step in optimizing the biosensor was to evaluate how the nature of the capture probe affected the electrochemical signal. Two probes that mimic miR-155 were tested: a DNA oligonucleotide and an RNA oligonucleotide, each with an MB tag at the 3′ end as an electroactive reporter. As shown in Figure 2A, both probes produced a similar “signal-off” response after hybridization with LNA-anti-miR-155, confirming that both could serve as effective recognition elements. For the subsequent experiments, the RNA probe was selected, not because it offered superior electrochemical performance, but because its structural similarity to native miRNA makes it more biologically representative of the natural antisense interaction of LNA oligonucleotides. LNA antisense oligonucleotides are designed to hybridize with endogenous miRNAs, and LNA monomers adopt an RNA-like (C3′-endo) sugar conformation that favors A-type duplex formation with RNA targets.^21^ Thus, using an RNA capture strand that mirrors the sequence of miR-155 provides a recognition interface that more closely reflects the intended pharmacological interaction. However, since the DNA probe demonstrated similar electrochemical behavior, it may represent a more robust alternative in applications where long-term stability or nuclease resistance is critical.

**Figure 2:**
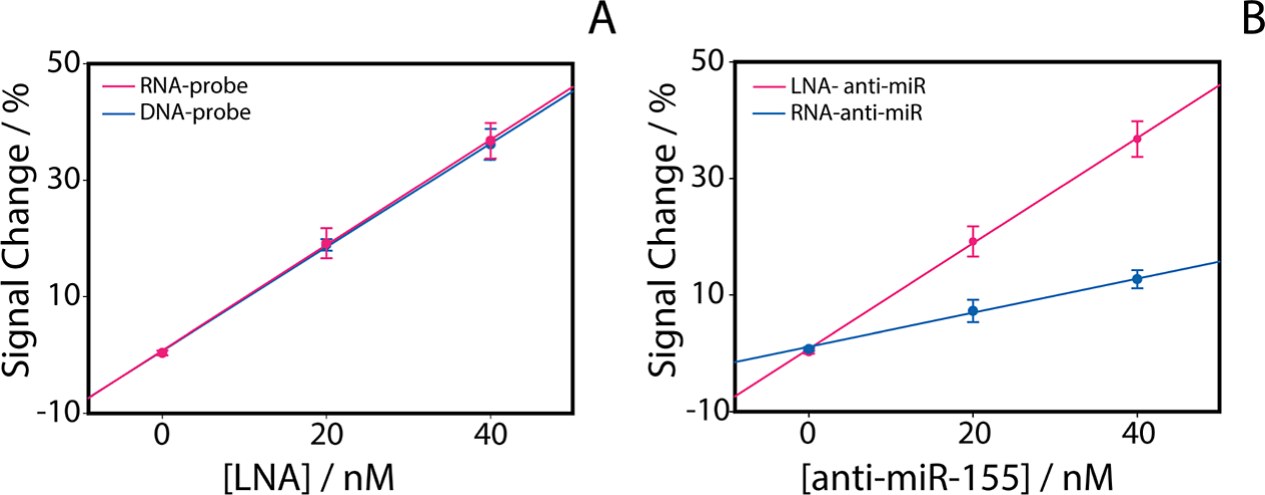
Optimization of the electrochemical biosensor for the detection of LNA-anti-miR-155. (A) Comparison between DNA and RNA capture probes with increasing concentrations of LNA-anti-miR-155. (B) Comparison between RNA and LNA versions of the anti-miR-155 target. In both experiments, the probe concentration was 100 nM, while the target concentrations (RNA and LNA) were 20 nM and 40 nM. All experiments were performed in five replicates.

As shown in Figure 2B, we performed control experiments comparing the biosensor response to the anti-miR-155 target in its RNA and LNA forms. Both targets hybridized with the RNA probe immobilized on the surface and produced a measurable signal-off response. However, the current decrease induced by the LNA target was consistently more pronounced than that of the RNA version. This difference reflects the structural advantage of LNA: biophysical studies have shown that incorporating LNA monomers typically increases the melting temperature of RNA-containing duplexes by about 2–8 °C per LNA residue and stabilizes the duplex free energy by roughly 1–1.5 kcal mol^–1^ per substitution, indicating a marked gain in binding affinity and hybrid stability.^1,21,22^ In our system, this stronger and more stable interaction with the single-stranded RNA probe translates into formation of a more rigid duplex that increases the distance of the methylene blue tag from the electrode surface. This enhanced interaction explains why LNA is used as a therapeutic antisense molecule, as its structure binds and inhibits the endogenous miRNA target more effectively.^23,24^

To maximize the analytical response of the biosensor toward LNA-anti-miR-155, we carried out a systematic optimization of three key parameters: the volume of AuNPs deposited on the electrode, the surface density of the capture probe, and the SWV frequency used for electrochemical readout. A full account of these studies is provided in the Supporting Information (Figure S2). The AuNP layer is essential for enhancing electron transfer and providing anchoring sites for the thiolated RNA probe. In addition, AuNP modification increases the effective electroactive surface area and creates a nanostructured, highly conductive interface, which results in higher methylene-blue baseline currents, improved signal-to-noise ratio, and faster electron transfer.^25^ As illustrated in Figure S2A, electrodes treated with 2 μL of AuNP suspension produced weak and unstable currents, consistent with incomplete nanoparticle coverage. In contrast, applying 8–12 μL generated nonuniform layers that occasionally detached during rinsing, likely due to loosely aggregated particles and uneven drying. A 4 μL deposition achieved the most favorable balance, forming a uniform and adherent coating that yielded stable baseline currents and the largest percentage signal change; this condition was therefore selected for all subsequent experiments. The next step involved adjusting the probe density to optimize signal intensity and reproducibility (Figure S2B). Immobilization at 50 nM produced a very low baseline current, owing to the limited number of electroactive MB tags on the surface, and the relative signal change after hybridization was not sufficiently reproducible. Increasing the probe concentration to 100 nM enhanced the baseline current and produced consistent signal-off responses. At higher surface loadings (≥250 nM), the current response became more variable, and the signal-off effect diminished, likely due to steric crowding and electrostatic repulsion hindering hybridization.^26^ Based on this evaluation, 100 nM was identified as the optimal probe density. Finally, the effect of SWV frequency on the biosensor response was evaluated (Figure S2C). A frequency of 25 Hz provided the clearest and most reproducible signal changes, characterized by sharp peaks and favorable signal-to-noise ratios: at 10 Hz, the current was stable but low, with broad peaks that limited sensitivity, whereas frequencies of 50–100 Hz increased the baseline current but introduced noise and reduced the reliability of hybridization-induced quenching. To quantitatively support the choice of 25 Hz, the frequency dependence was further evaluated by one-way ANOVA on the signal change at 40 nM LNA-anti-miR-155 followed by Tukey’s HSD test (family wise error rate = 0.05). This analysis indicated that 10 Hz afforded the strongest statistical discrimination, but that 25 Hz was also significantly different from the higher frequencies (50 and 100 Hz); in light of its superior measurement reproducibility and more favorable peak shape compared with 10 Hz, 25 Hz was selected as the operating frequency for all analytical measurements. The corresponding histogram with significance markers is shown in Figure S3 of the Supporting Information.

After covering the sensing area with a fixed concentration of LNA target (1 nM), the probe–target interaction time was systematically investigated using a probe density of 100 nM. The study was carried out over an incubation-time range up to 40 min, as shown in Figure S4A of the Supporting Information, monitoring how the analytical signal evolved as hybridization progressed. Under these conditions, an incubation time of 30 min provided a satisfactory compromise between sensitivity, repeatability, and overall analysis time, since prolonged incubation did not produce a statistically significant further change in the signal.

### Analytical Characterization in Standard and Human Plasma

After completing the optimization of all experimental parameters, the analytical performance of the electrochemical biosensor for the detection of LNA-anti-miR-155 was systematically evaluated. The study was conducted by increasing the target concentration over a wide range, from 0.01 to 1000 nM, while maintaining all previously established optimized conditions.

As shown in Figure 3A, the calibration curve and representative SWV responses in the buffer revealed a progressive decrease in current with increasing concentrations of LNA-anti-miR-155, consistent with the expected signal-off mechanism.

**Figure 3:**
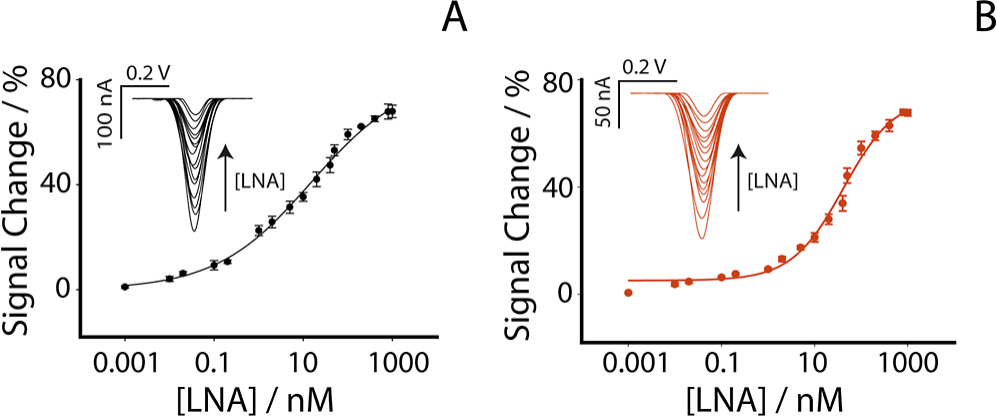
(A) Calibration curve and SWV curves obtained in buffer solution by testing different concentrations of LNA-anti-miR-155 from 0.01 to 1000 nM. (B) Calibration curve and SWV curves obtained in human plasma by testing different concentrations of LNA-anti-miR-155 from 0.01 to 1000 nM. All experiments were performed in five replicates. SWV parameters: *t* eq = 5 s, *E* start = 0.0 V, *E* end = −0.6 V, *E* step = 0.001 V, amplitude = 0.01 V, frequency = 25 Hz.

Figure 3B shows the corresponding calibration in undiluted human plasma, where the same trend was observed despite the presence of potential interferents. In both matrices, the response followed a semilogarithmic sigmoidal profile, typical of surface-confined hybridization events and are well described by a four parameter Hill (logistic) function rather than by a single linear regression.^27^ This trend reflects the gradual saturation of the immobilized RNA probe mimicking miRNA-155 as the concentration of LNA-anti-miR-155 increases, resulting in the formation of a more rigid RNA/LNA duplex that limits the mobility of the MB tag and reduces electron transfer. For nonlinear sigmoidal calibrations, several methodological studies recommend defining the limit of detection (LOD) directly on the adapted logistic/Hill model, which guarantees a response clearly superior to the variability of the blank, instead of forcing a global linear approximation. In line with this approach, and consistently with previous work on surface-based biosensors, we defined the limit of detection as the concentration of LNA-anti-miR-155 that produces a 10% change of the total signal between the baseline (no target) and the saturation region of the Hill fit.,^28–30^ Using this approach, the LOD in buffer was 40 pM, while the LOD in plasma was 300 pM, confirming that the biosensor maintains subnanomolar sensitivity even in complex, untreated biological matrices. This sensitivity is particularly relevant in the context of therapeutic monitoring, as pharmacokinetic studies of LNA-based antisense oligonucleotides consistently report circulating plasma concentrations in the low-nanomolar to submicromolar range following systemic administration. Notably, peak plasma levels are typically observed between a few nanomolar and several hundred nanomolar, with trough concentrations often remaining above 1 nM.^12,31,32^ The ability of the sensor to detect LNA targets at 300 pM, well below the lower end of this therapeutic range, supports its potential applicability for tracking antisense drug levels across the full pharmacokinetic profile. To provide a quantitative illustration of matrix effects, we compared the signal change at a representative concentration (20 nM LNA-anti-miR-155) in buffer and undiluted plasma. Under these conditions, the mean signal change decreases from 43.4 ± 1.4% in buffer to 29.0 ± 1.7% in plasma, corresponding to an absolute loss of ∼14 percentage points and retention of approximately 67% of the buffer response. This attenuation and the reduced slope of the plasma calibration curve are consistent with matrix effects commonly observed when translating electrochemical nucleic acid sensors from ideal buffer to undiluted biological fluids, where proteins, lipids, and other macromolecules can foul the interface, reduce probe accessibility, and alter local ionic strength and dielectric properties, thereby compressing the dynamic range, especially at lower target concentrations.^33,34^ To evaluate the precision of the approach, we initially measured the response of five independently fabricated electrodes at 20 nM LNA-anti-miR-155 within a single fabrication batch, obtaining a relative standard deviation (RSD %) of 6% in buffer and 7% in undiluted human plasma, which reflects intrabatch precision. To further assess reproducibility, we fabricated a second, independent batch of paper-based AuNP-modified electrodes and repeated the measurements under identical conditions. Under these conditions, the interbatch RSD between the two fabrication batches was 5% in buffer and 6% in plasma at 20 nM, indicating that different batches of paper-based electrodes yield highly comparable signal-off responses in both matrices and that the platform is precise both within and across fabrication batches.

To independently verify sequence selectivity under well-controlled conditions, we also evaluated the response in the presence of four endogenous human miRNAs (miR-21, miR-218, miR-29c-5p, and miR-101-5p) and an antisense LNA oligonucleotide targeting miR-644a, all noncomplementary to the capture probe, tested at the same concentration as the target (1 nM; Figure S4B, Supporting Information). Under these conditions, 1 nM LNA-anti-miR-155 produced a clear signal change, whereas 1 nM of each interferent did not induce any measurable response, indicating that the sensor signal is governed by specific hybridization rather than nonspecific adsorption or cross-reactivity. In addition, the mixed monolayer of thiolated RNA probe and 6-mercapto-1-hexanol, together with operation in the concentration-limited hybridization regime and the good baseline stability observed in the absence of target, helps minimize signal losses unrelated to specific duplex formation, which mitigates the risk of false-positive responses that can affect signal-off architectures.^35^ Overall, these results demonstrate that the optimized biosensor enables ultrasensitive, selective, and reproducible detection of LNA-based antisense oligonucleotides, with the robustness required for potential therapeutic monitoring applications.

In order to validate the developed system, spiked plasma samples containing known concentrations of LNA-anti-miR-155 in the 0.1–800 nM range were analyzed in parallel by the paper-based electrochemical biosensor and by a solution-phase RiboGreen fluorescence assay for oligonucleotide quantification. Spiked plasma samples were analyzed by both methods, and the apparent recoveries are summarized in Table 1, where the biosensor shows recoveries of approximately 94–106% over the entire range, in line with commonly accepted analytical criteria for accuracy and in close agreement with the RiboGreen values.

**Table 1:** Correlation Among RiboGreen Assay and the Paper Screen-Printed Electrodes of Various LNA-Anti-miR-155-Spiked Plasma Concentrations and Apparent Recoveries of the Two Methods

spiked concentration (nM)	ribogreen assay (nM)	ribogreen assay app. recovery (%)	SPE (nM)	SPE app. recovery (%)	correlation (%)
0.1	0.11	110	0.098	98	80
5	4.5	90	4.7	94	104
10	9.1	91	9.1	91	100
20	23	115	19.13	95	83
40	39.14	98	41.4	103	105
100	95	95	106	106	155
200	211	105	199.8	99	95
400	372.7	93	410	102	110
800	770	96	827	103	107

Statistical evaluation of the paired data (Figure 4) revealed excellent agreement between the two approaches, with a Pearson correlation coefficient *r* = 0.999 (*p* < 0.001), a coefficient of determination *R* ^2^ = 0.998, and a regression slope of 0.93 ± 0.01, indicating that 99.8% of the variance in the RiboGreen measurements is explained by the biosensor results. These findings demonstrate that the proposed sensor provides quantitatively consistent results with an independent fluorescence-based assay, supporting its suitability for reliable determination of therapeutic LNA antisense oligonucleotides in plasma.

**Figure 4:**
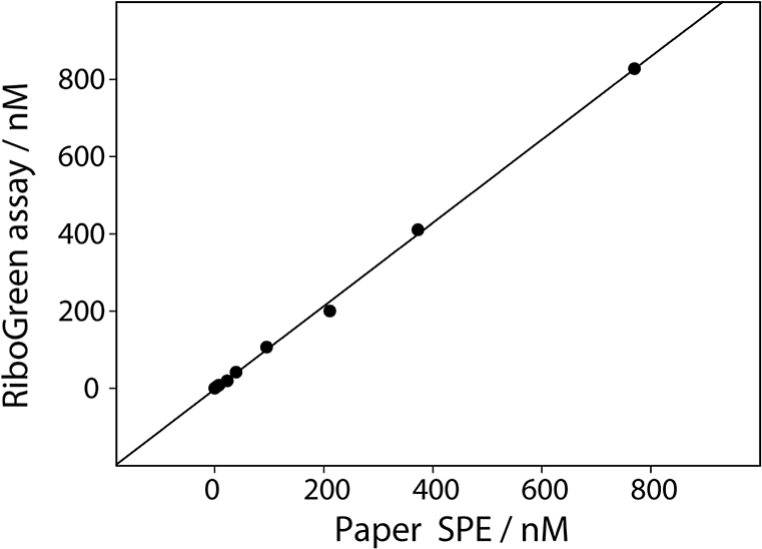
Correlation between LNA-anti-miR-155 concentrations measured using the RiboGreen assay and the paper based electrochemical biosensor in spiked plasma samples. The black line represents the linear regression fit (*y* = 0.93*x* + 2.75, *R* ^2^ = 0.998).

Beyond these analytical figures of merit, it is important to consider how the platform is intended to operate in practice. The device is designed as a single-use strip without surface regeneration, which is in line with most electrochemical point-of-care tests and helps avoid carry-over while supporting low-cost paper fabrication, even though it does not provide continuous monitoring on the same electrode. In this context, the assay offers a practical sample-to-result time of about 35–40 min, including 30 min of hybridization and a few minutes for rinsing and SWV readout, which is compatible with near-patient pharmacokinetic checks and considerably shorter than the multihour workflows of LC–MS/MS or qPCR. In its present form, the platform is intended for intermittent plasma measurements at selected time points, rather than continuous or implantable monitoring, and is best suited to near-patient or point-of-care-compatible use once plasma has been obtained by simple centrifugation.

## Conclusions

In this work, a paper-based electrochemical biosensor was designed, optimized, and applied for the direct detection of LNA-based antisense oligonucleotides, using LNA-anti-miR-155 as a therapeutic target model. The detection platform consists of a screen-printed electrode modified with gold nanoparticles and functionalized with an RNA probe that mimics miRNA-155, enabling highly selective hybridization and a signal-off electrochemical response. After comprehensive optimization of probe density, AuNP deposition volume, and SWV acquisition parameters, and hybridization time, the biosensor demonstrated ultrasensitive detection in a range of 0.01–1000 nM, with detection limits of 40 pM in buffer and 300 pM in undiluted human plasma, while maintaining good precision (RSD 6% in buffer, 7% in plasma) and sub nanomolar sensitivity under stringent matrix conditions. Selectivity studies with noncomplementary endogenous miRNAs and an off-target antisense LNA, together with operation in undiluted plasma without pretreatment, confirmed that the response is governed by specific hybridization rather than nonspecific adsorption, and that matrix effects mainly attenuate signal amplitude rather than compromising analytical performance. Orthogonal validation against a RiboGreen fluorescence assay in spiked plasma showed apparent recoveries of about 94–106% and an excellent correlation, supporting the quantitative reliability of the electrochemical platform for therapeutic monitoring. In its current configuration, the device operates as a single-use, reagentless, amplification-free strip assay with a sample-to-result time of approximately 35–40 min, making it well suited to short-turnaround, near-patient assessment of circulating antisense LNA drug levels rather than continuous monitoring. Overall, this study establishes a robust, low-cost, and portable paper-based electrochemical platform for direct detection of intact antisense LNA oligonucleotides in undiluted plasma, highlighting its potential as a point-of-care-compatible tool to support personalized dosing and pharmacokinetic monitoring in precision medicine.

## Supplementary Material



## Notes

UV–vis absorption spectrum of the citrate-stabilized gold nanoparticles synthesized by the Turkevich method, recorded between 300 and 700 nm in water. Optimization of electrode surface and electrochemical parameters for the detection of LNA-anti-miR-155. Frequency study. Statistical analysis was conducted using ANOVA followed by Tukey’s HSD test (FWER = 0.05). Study of hybridization kinetics up to 40 minutes using a probe density of 100 nM and 1 nM of LNA-anti-miR-155. Paper-based screen-printed electrode fabrication and wax patterning protocol; gold nanoparticle synthesis and characterization; optimization of AuNP deposition volume, probe density, and SWV frequency; statistical analysis of frequency dependence; hybridization kinetics and selectivity studies (Figures S1–S4) (PDF)
