# Supplementary material for: Anticancer LNA Oligonucleotides Detection through a Simple Paper-Based Platform
Source: ACS Meas Sci Au. 2026 Feb 10;6(2):374–81. doi: 10.1021/acsmeasuresciau.5c00164 (PMC13087959; doi:10.1021/acsmeasuresciau.5c00164)
Supplement: Supplementary file 1 [file tg5c00164_si_001.pdf]

Supporting Information

Anticancer LNA oligonucleotides detection through a simple paper-based platform

Ada Raucci[1,4,⊥,*], Giovanna Liciberto[1,⊥], Michele Guida[1], Thomas Lee Moore[1], Canio Martinelli[2,3], Michelino De Laurentiis[4], Antonio Giordano[2,5], Stefano Cinti[1,2,6*]

[1] University of Naples Federico II, Department of Pharmacy, Via D. Montesano 49, Naples, 80131, Italy

[2] Sbarro Institute for Cancer Research and Molecular Medicine, Center for Biotechnology, College of Science and Technology, Temple University, Philadelphia, PA, 19122, USA

[3] Department of Human Pathology of Adult and Childhood "Gaetano Barresi", Unit of Obstetrics and Gynecology, University of Messina, Via Consolare Valeria 1, Messina, 98124, Italy

[4] Department of Breast and Thoracic Oncology, Istituto Nazionale Tumori IRCCS "Fondazione G. Pascale", Napoli, Italy

[5] Department of Medical Biotechnologies, University of Siena, 53100 Siena, Italy

[6] Department of Chemistry, Faculty of Science, Chulalongkorn University, Bangkok 10330, Thailand.

[*]Corresponding authors.

E-mail addresses: ada.raucci@unina.it, stefano.cinti@unina.it

Paper-based screen-printed electrode (SPEs) preparation

A three-electrode system was manually fabricated using a screen-printing technique according to protocols previously described in the literature.[1,2] Biosensing devices were fabricated on standard office paper (Fabriano, 80 g/m²), chosen for its low cost and compatibility with wax patterning. Initially, a hydrophobic layer was defined by digitally drawing the fluidic geometry in Adobe Illustrator, which was then printed on the paper using a ColorQube 8580 solid ink printer (Xerox, USA). To allow the wax to permeate the paper matrix and create effective fluidic boundaries, the printed sheets were heat-treated at 100°C for one minute. This step ensured the formation of well-defined hydrophobic barriers.[3] Next, the electrochemical elements were deposited by screen printing using two separate masks: one designated for silver/silver chloride ink (reference electrode) and the other for carbon ink (working electrodes and counter electrodes). Both conductive inks were obtained from SunChemical (USA). After deposition, each printed layer was thermally cured at 60°C for 30 minutes to ensure ink adhesion and conductivity.

AuNPs synthesis

AuNPs were prepared according to a modified protocol inspired by previous literature.[4] Prior to synthesis, all glassware and magnetic stirring equipment were thoroughly decontaminated to remove

potential impurities. This was accomplished by sequential immersion in freshly prepared aqua regia (a 3:1 v/v mixture of hydrochloric and nitric acid), followed by extensive rinsing with distilled water. A second cleaning step involved treatment with piranha solution (7:3 v/v H_2SO_4/H_2O_2), after which the objects were again thoroughly rinsed and allowed to dry. For AuNPs synthesis, the reaction was conducted at room temperature using a laboratory reaction vessel. Specifically, 9 mL of distilled water was mixed with 1 mL of a 0.01 g/mL solution of chloroauric acid ($HAuCl_4$), followed by the addition of 2 mL of sodium citrate solution at the same concentration. To initiate nucleation and reduce the gold precursor, 0.5 mL aliquots of freshly prepared 20 mM sodium borohydride were added dropwise under continuous stirring. The reaction was maintained under dark conditions with gentle stirring overnight to ensure complete formation of the nanoparticles. The obtained colloidal AuNP suspension was stored at 4 °C until further use. Beyond the synthetic protocol, the concentration of the AuNP suspension used for electrode modification was quantified from its optical properties. Citrate‐stabilized AuNPs synthesized by this Turkevich‐type protocol exhibited a

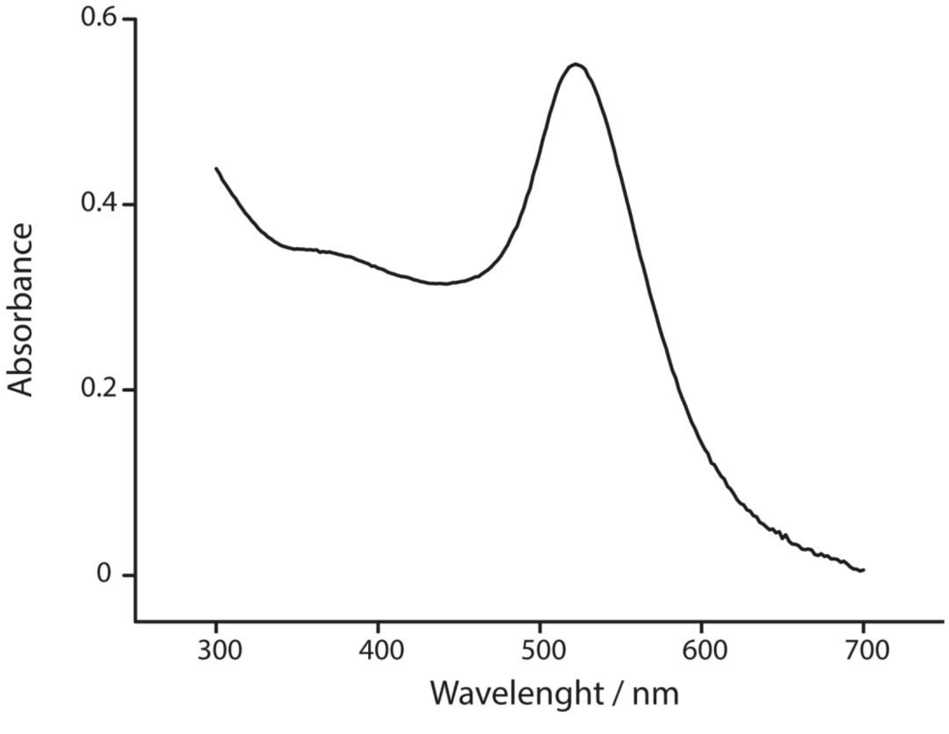

localized surface plasmon band at 522–524 nm; a 5‐fold diluted suspension showed an absorbance of 0.552 at this wavelength (1 cm path length), as reported in the Figure S1.

Figure S1. UV–Vis absorption spectrum of the citrate-stabilized gold nanoparticles synthesized by the Turkevich method, recorded between 300 and 700 nm in water.

Assuming a particle diameter of ~20 nm, consistent with our previous characterization of AuNPs obtained with the same synthesis,[5] and using size‐dependent extinction coefficients for

citrate-stabilized spherical AuNPs at the surface plasmon resonance maximum as reported by Haiss et al.,[6] the nanoparticle concentration was estimated via the Beer–Lambert law to be approximately 3 nM for the undiluted stock. Under these conditions, the optimized deposition volume of 4 µL corresponds to roughly 12 fmol of AuNPs per working electrode.

Optimization of experimental parameters

After optimizing the molecular recognition step, the next step involved modifying the working electrode with AuNPs, which play a crucial role in improving electron transfer and providing a high-affinity surface for immobilizing the thiolated RNA probe. In addition, AuNP modification increases the effective electroactive surface area and creates a nanostructured, highly conductive interface, which results in higher methylene-blue baseline currents, improved signal-to-noise ratio, and faster electron transfer [7]. The effect of the deposited volume was systematically evaluated using 2, 4, 8, and 12 µL of AuNPs suspension, as shown in Fig. S2.A. Electrodes modified with 2 µL showed weak and unstable signals, consistent with insufficient nanoparticle coverage and a limited active surface area. In contrast, higher volumes of 8 µL and 12 µL led to uneven coatings and occasional delamination during rinsing, probably due to the formation of weakly bound aggregates and uneven drying. Among the conditions tested, 4 µL produced a homogeneous and adherent AuNPs layer, providing the most reproducible baseline currents and maximum signal intensity, and were therefore selected as the optimal deposition volume for subsequent experiments.

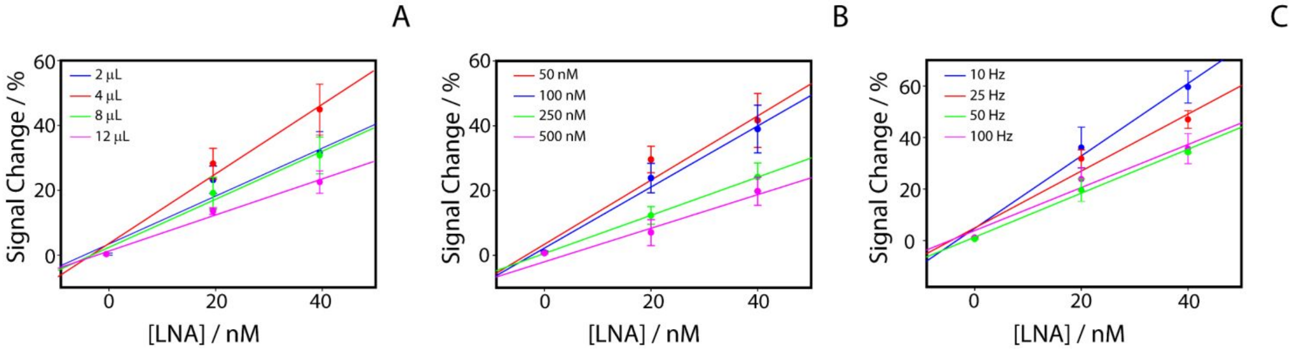

Figure S2. Optimization of electrode surface and electrochemical parameters for the detection of LNA-anti-miR-155. (A) Percentage signal change for LNA-anti-miR-155 at 20 nM and 40 nM using different volumes of AuNP deposition on the working electrode (2, 4, 8, and 12 µL). (B) Percentage signal change obtained with different probe densities (50, 100, 250, and 500 nM). (C) Effect of SWV frequency (10, 25, 50, and 100 Hz) on the electrochemical response. All experiments were performed in five replicates.

Once the AuNP layer was optimized, the effect of the probe surface density on biosensor performance was studied, Fig. S2.B. The capture probe (miR-155) was immobilized at concentrations ranging from 50 to 500 nM. At the lowest density (50 nM), the baseline current was significantly reduced due to

the limited number of electroactive methylene blue tags on the electrode surface. Interestingly, this configuration produced a slightly higher relative signal change following LNA-anti-miR-155 hybridization, although the absolute current was too low to ensure robust reproducibility. Increasing the probe concentration to 100 nM resulted in a higher and more stable baseline current, with a signal quenching response comparable to that observed at 50 nM. At very high probe loads (250-500 nM), signal variation decreased and variability increased, likely due to steric hindrance and partial electrostatic repulsion, which hindered efficient hybridization. Therefore, an optimal probe density of 100 nM was selected, offering the best compromise between reproducibility, baseline current intensity, and reliable signal quenching behavior.[8] After establishing the optimal probe density, the final stage of biosensor optimization focused on electrochemical measurement parameters, particularly SWV frequency, which directly affects signal intensity, peak shape, and reproducibility (Fig. S2.C). Frequencies of 10, 25, 50, and 100 Hz were tested to evaluate their influence on the electrochemical response of the MB-labeled probe. At low frequency (10 Hz), the current response was stable but relatively low, and the peaks were broader, reducing sensitivity. Increasing the frequency to 25 Hz resulted in the highest and most reproducible signal change, with well-defined peaks and a favorable signal-to-noise ratio. At higher frequencies (50-100 Hz), the baseline current increased, but the signal change became less reliable due to noise and reduced hybridization-induced peak shifts. To quantitatively support the choice of 25 Hz, the frequency dependence was further evaluated by one-way ANOVA on the signal change at 40 nM LNA-anti-miR-155 followed by Tukey's HSD test (family-wise error rate = 0.05). This analysis indicated that 10 Hz afforded the strongest statistical discrimination, but that 25 Hz was also significantly different from the higher frequencies (50 and 100 Hz); in light of its superior measurement reproducibility and more favourable peak shape compared with 10 Hz, 25 Hz was selected as the operating frequency for all analytical

measurements. The corresponding histogram with significance markers is shown in Figure S3.

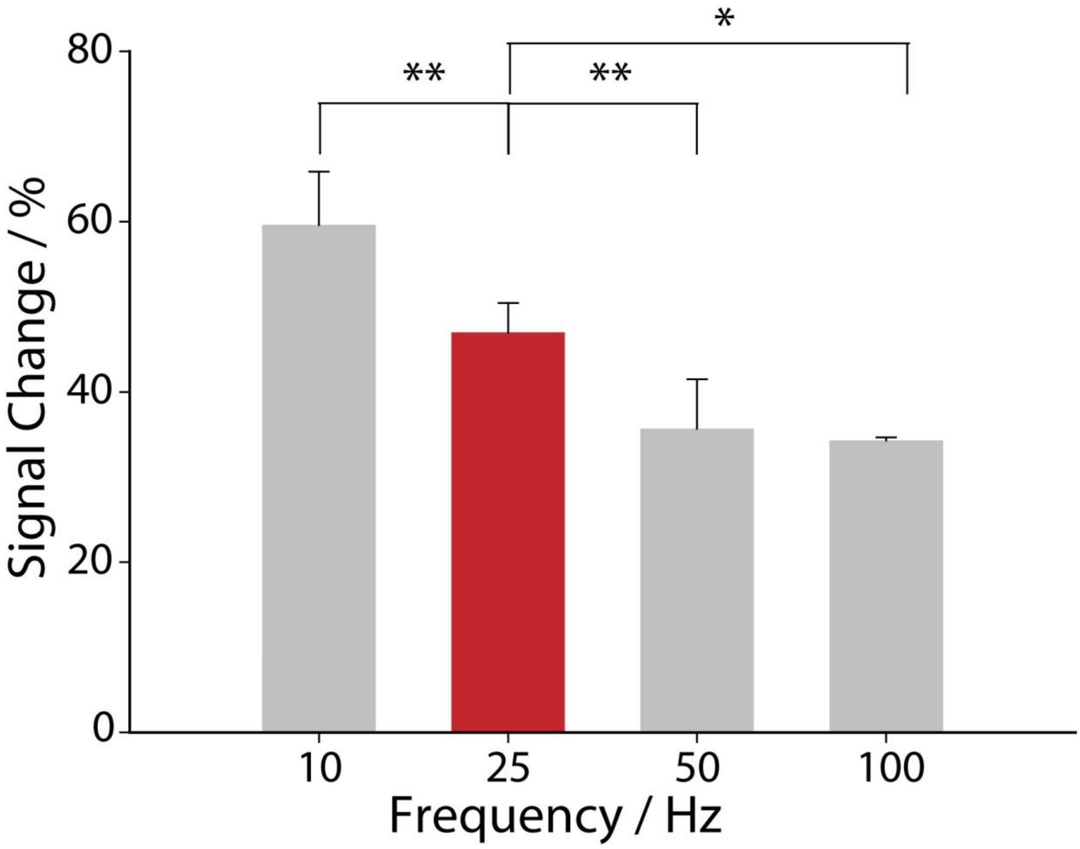

Figure S3. Frequency study. Statistical analysis was conducted using ANOVA followed by Tukey's HSD test (FWER = 0.05). Horizontal lines indicate significant differences between groups: **$p < 0.01$, *$p < 0.05$.

Study of hybridization kinetics and selectivity

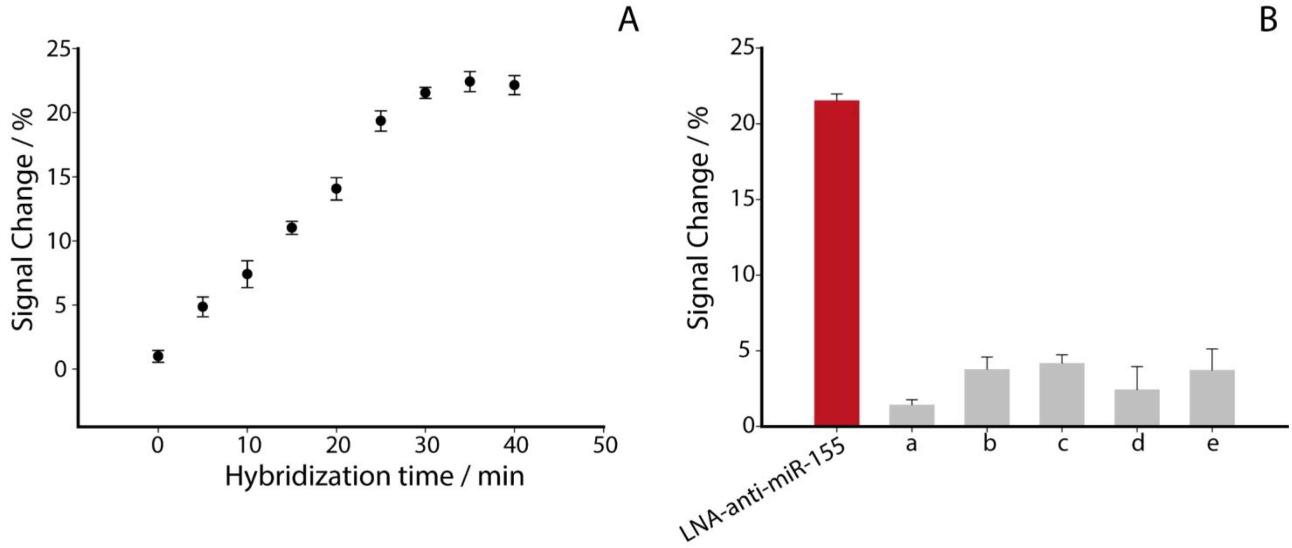

Figure S4. A) Study of hybridization kinetics up to 40 minutes using a probe density of 100 nM and 1 nM of LNA-anti-miR-155. B) Selectivity studies comparing the signal changes obtained in the presence of four endogenous human miRNAs (a,b,c,d) and an antisense oligonucleotide LNA targeting miRNA-644a (e). All experiments were performed in five replicates.

REFERENCES

(1) Raucci, A.; Cimmino, W.; Grosso, S. P.; Normanno, N.; Giordano, A.; Cinti, S. Paper-Based Screen-Printed Electrode to Detect miRNA-652 Associated to Triple-Negative Breast Cancer. *Electrochimica Acta* **2024**, *487*, 144205. https://doi.org/10.1016/j.electacta.2024.144205.

(2) Raucci, A.; Sorrentino, G.; Singh, S.; Borbone, N.; Oliviero, G.; Piccialli, G.; Terracciano, M.; Cinti, S. Cost-Effective, User-Friendly Detection and Preconcentration of Thrombin on a Sustainable Paper-Based Electrochemical Platform. *Anal Bioanal Chem* **2025**, *417* (9), 1863–1872. https://doi.org/10.1007/s00216-025-05764-9.

(3) Cinti, S.; Moscone, D.; Arduini, F. Preparation of Paper-Based Devices for Reagentless Electrochemical (Bio)Sensor Strips. *Nat Protoc* **2019**, *14* (8), 2437–2451. https://doi.org/10.1038/s41596-019-0186-y.

(4) Iula, G.; Raucci, A.; Ratti, F.; Darwish, I. A.; Cinti, S. Smart Electrochemical Sensing for Rapid Detection of Iron in Dietary Supplements. *J. Electrochem. Soc.* **2025**. https://doi.org/10.1149/1945-7111/adbd7e.

(5) Miglione, A.; Raucci, A.; Amato, J.; Marzano, S.; Pagano, B.; Raia, T.; Lucarelli, M.; Fuso, A.; Cinti, S. Printed Electrochemical Strip for the Detection of miRNA-29a: A Possible Biomarker Related to Alzheimer's Disease. *Anal. Chem.* **2022**, *94* (45), 15558–15563. https://doi.org/10.1021/acs.analchem.2c03542.

(6) Haiss, W.; Thanh, N. T. K.; Aveyard, J.; Fernig, D. G. Determination of Size and Concentration of Gold Nanoparticles from UV−Vis Spectra. *Anal. Chem.* **2007**, *79* (11), 4215–4221. https://doi.org/10.1021/ac0702084.

(7) Hu, K.; Lan, D.; Li, X.; Zhang, S. Electrochemical DNA Biosensor Based on Nanoporous Gold Electrode and Multifunctional Encoded DNA−Au Bio Bar Codes. *Anal. Chem.* **2008**, *80* (23), 9124–9130. https://doi.org/10.1021/ac8017197.

(8) Esteban Fernández de Ávila, B.; Watkins, H. M.; Pingarrón, J. M.; Plaxco, K. W.; Palleschi, G.; Ricci, F. Determinants of the Detection Limit and Specificity of Surface-Based Biosensors. *Anal. Chem.* **2013**, *85* (14), 6593–6597. https://doi.org/10.1021/ac4012123.
